# Comparison of replica leaf surface materials for phyllosphere microbiology

**DOI:** 10.1371/journal.pone.0218102

**Published:** 2019-06-06

**Authors:** Rebecca Soffe, Nicola Altenhuber, Michal Bernach, Mitja N.P. Remus-Emsermann, Volker Nock

**Affiliations:** 1 Department of Electrical and Computer Engineering, University of Canterbury, Christchurch, New Zealand; 2 School of Biological Sciences, University of Canterbury, Christchurch, New Zealand; Tallinn University of Technology, ESTONIA

## Abstract

Artificial surfaces are routinely used instead of leaves to enable a reductionist approach in phyllosphere microbiology, the study of microorganisms residing on plant leaf surfaces. Commonly used artificial surfaces include, flat surfaces, such as metal and nutrient agar, and microstructured surfaces, such as isolate leaf cuticles or reconstituted leaf waxes. However, interest in replica leaf surfaces as an artificial surface is growing, as replica surfaces provide an improved representation of the complex topography of leaf surfaces. To date, leaf surfaces have predominantly been replicated for their superhydrophobic properties. In contrast, in this paper we investigated the potential of agarose, the elastomer polydimethylsiloxane (PDMS), and gelatin as replica leaf surface materials for phyllosphere microbiology studies. Using a test pattern of pillars, we investigated the ability to replicate microstructures into the materials, as well as the degradation characteristics of the materials in environmental conditions. Pillars produced in PDMS were measured to be within 10% of the mold master and remained stable throughout the degradation experiments. In agarose and gelatin the pillars deviated by more than 10% and degraded considerably within 48 hours in environmental conditions. Furthermore, we investigated the surface energy of the materials, an important property of a leaf surface, which influences resource availability and microorganism attachment. We found that the surface energy and bacterial viability on PDMS was comparable to isolated *Citrus × aurantium* and *Populus × canescens* leaf cuticles. Hence indicating that PDMS is the most suitable material for replica leaf surfaces. In summary, our experiments highlight the importance of considering the inherent material properties when selecting a replica leaf surface for phyllosphere microbiology studies. As demonstrated, a PDMS replica leaf offers a control surface that can be used for investigating microbe-microbe and microbe-plant interactions in the phyllosphere, which will enable mitigation strategies against pathogens to be developed.

## 1 Introduction

Many microorganisms thrive on plants. Microorganisms reside either permanently or temporarily in the plant environment and, they contribute to the health of the plant host. These microorganisms live in three interconnected compartments: the spermosphere, rhizosphere, and phyllosphere [1–6]. To date research has primarily been focused on the rhizosphere [6, 7]. However recently, phyllosphere microbiology, the study of microorganisms which reside on plant leaf surfaces, has gained increasing interest. This increase is attributed to the growing awareness of the role that microorganisms in the phyllosphere have on the health of the plant host. Microorganisms in the phyllosphere are in direct contact with the plant cuticle. The plant cuticle, is a protective waxy film that coats the leaves of plants and prevents pathogenic attacks against the plant host [8]. In addition, the cuticle prevents water, ion, and nutrient loss [9].

Leafy greens, such as those grown for human consumption (*i*.*e*. lettuce, rhubarb, and parsley), are exposed to a range of potential contamination sources [4–7, 10]. These can include: irrigation water, soil, fertiliser, farm workers, and equipment used around a farm [11, 12]. Such unwanted contamination can result in unwanted microorganisms entering the phyllosphere. This can cause diseases that are harmful to humans or detrimental to plant health. This is of particular concern as leafy greens are often consumed raw or with minimal processing, which does not remove or kill unwanted contamination [4, 10–14]. In some cases, contamination can lead to outbreaks that can cause serious illnesses [15–17]. Unwanted leaf contamination is of an increased concern with increasing produce demand, large-scale production and distribution. Consequently, further studies to understand phyllosphere microbiology are imperative for developing mitigation strategies. Potential mitigation strategies may include: introducing other microorganisms to prevent against pathogens detrimental to either the plant host or humans; or developing different cleaning protocols for leafy greens [17, 18].

The use of artificial surfaces is common practice in phyllosphere microbiology. Artificial surfaces are used instead of a living leaf to enable a reductionist approach. Such an approach, enables the identification of individual factors influencing microorganism function and viability in a controlled environment, for example, to study the influence of contamination of leafy greens [13][19, 20]. Artificial surfaces used in phyllosphere microbiology can be classified due to their lateral heterogeneity as either flat or microstructured.

A flat surface is defined as a surface that is laterally homogenous. In general, a flat surface will have no lateral chemical or biological heterogeneity at the time of inoculation with microorganisms. Commonly used flat surfaces include nutrient agar or inert surfaces (for example, metal, plastic, and glass). Nutrient agar is commonly used to investigate the influence of different nutrient compositions on microorganism interactions and colonisations. For example, Jacobs *et al*. investigated the role of pigmentation, ultraviolet radiation tolerance and leaf colonisation strategies in epiphytic survival using nutrient agar [21]. Inert flat surfaces on the other hand, are commonly utilised to investigate the attachment processes of microorganisms. For example, Rivas *et al*. observed variation in surface attachment amongst strains of Shiga toxigenic *Escherichia coli* (*E*. *coli*) on stainless steel surfaces [22].

In contrast, microstructured surfaces are defined as surfaces which are laterally heterogeneous, and will generally have no lateral chemical or biological heterogeneity at the time of inoculation. A microstructured surface is more representative of the surface of the leaf, as the cuticle of a leaf is physically (and chemically) laterally heterogeneous [23]. Commonly used microstructured surfaces include reconstituted leaf wax, leaf peels, isolated leaf cuticles, and microfabricated surfaces [24–26]. For instance, Remus-Emsermann *et al*. examined the permeability of fructose through isolated poplar (*Populus × canescens*) cuticles, to understand microorganism growth patterns in the phyllosphere [27].

Although microstructured surfaces are suitable for their respective applications, they do not entirely represent the complex nature of the topography of plant leave surfaces [28, 29]. Recent studies have utilised double-casting protocols to overcome this limitation. As such replica leaf surfaces have been produced in agarose, dental wax, or polydimethylsiloxane (PDMS) [28–31], but predominately for their self-cleaning properties [32–34]. However, for phyllosphere microbiology studies are beginning to appear using microfabricated replica leaf surfaces. For example, Zhang *et al*. used double-casting to produce replica spinach leaf surfaces in agarose, and they investigated the interaction of *E*. *coli* on flat agarose and agarose replica leaf surfaces [31].

However, studies to date have not fully considered the potential influence of the replica leaf material on microorganism viability and behavior. The inherent properties of a material can influence microorganism behaviour, such that it is imperative that the suitability of a material as a replica leaf surface is characterized comprehensively. Investigations should include: (*i*) a resolution analysis to determine if the replica suitably mimics the complex topography of a leaf surface; (*ii*) examination of the degradation characteristics to determine if the replica leaf can maintain the topography throughout an experiment; and (*iii*) determine the hydrophobicity of a replica leaf material and how representative this is of a leaf surface. This is significant, as the hydrophobicity of the leaf surface can influence the attachment processes of microorganisms.

Given this existing knowledge gap, the purpose of our study was to determine a material suitable for producing replica leaf surfaces for phyllosphere microbiology. To that extent we investigated the suitability of three commonly used biocompatible materials: agarose, PDMS, and gelatin. Agarose has been used as a replica leaf surface, has well established microfabrication protocols, and is routinely used in phyllosphere microbiology studies [13, 31, 35]. PDMS, a silicone elastomer, is routinely used in biological applications. For example, PDMS is extensively use in lab-on-a-chip devices and in bioimprinting [36–42]. On the other hand, gelatin was initially used as a gelling agent for microbiology growth media, until it was succeeded by agar [43]. In addition, well established microfabrication protocols exist for gelatin [44].

For our investigation, we used a test pattern comprising of regular circular pillars comparable in size to microfeatures found on leaves. For each material we measured: (*i*) 3D optical surface profiles, (*ii*) the degradation of the materials over three days, and (*iii*) the hydrophobic properties in comparison to two generic isolated leaf cuticles (*Citrus × aurantium*, and *Populus × canescens*). In addition, to investigate the biological suitability of each material, we used a model leaf colonising bacterium *Pantoea agglomerans* 299R. We compared the bacteria survival results of the materials to bacterium on isolated *Citrus × aurantium* cuticles. We conjectured that out of the three materials we test, that PDMS is the most suitable replica leaf surface material for phyllosphere microbiology studies, as PDMS can exhibit nanoscale resolution and is stable in standard environmental conditions.

## 2 Materials and methods

**Fig 1:**
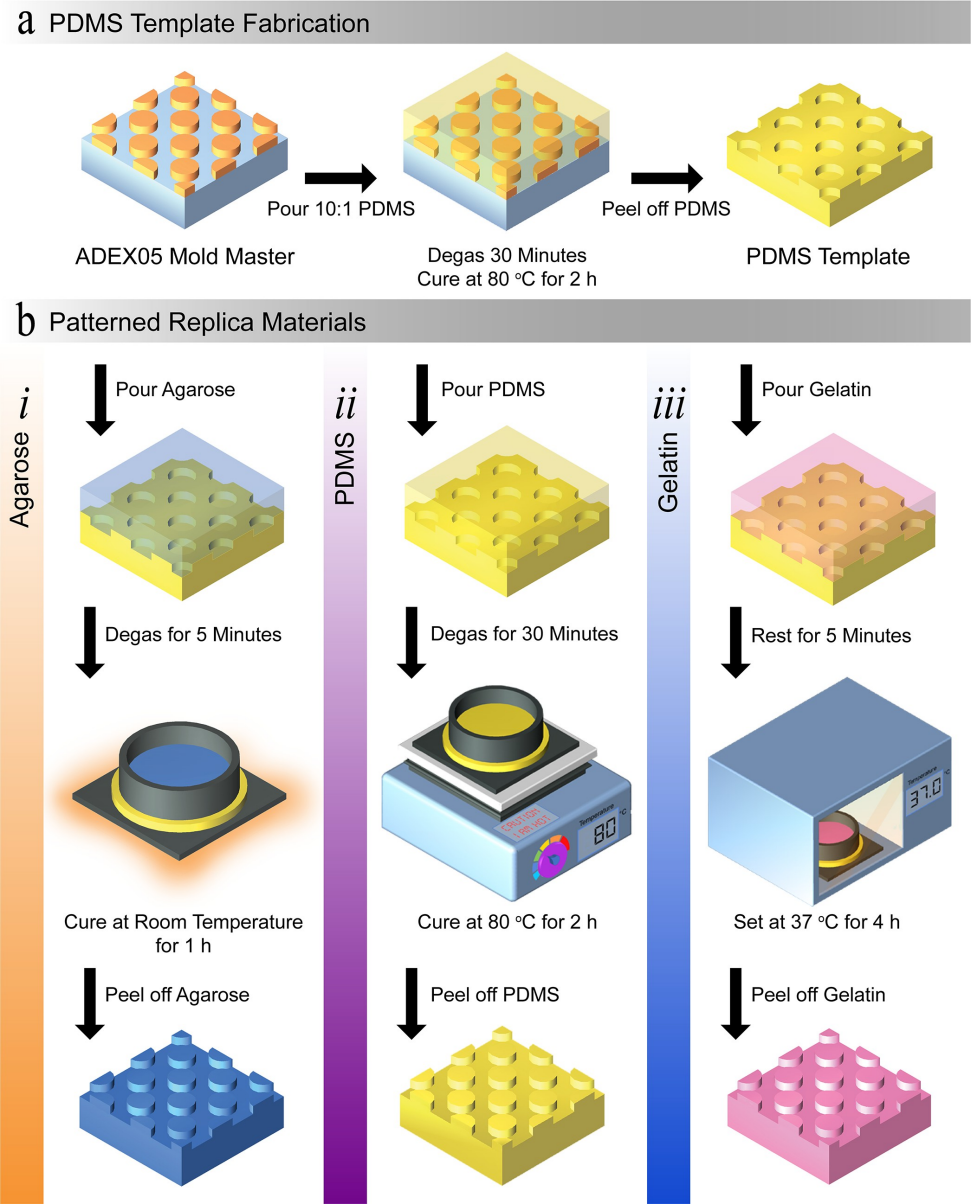
Schematic overview of the fabrication protocols. (a) Protocol used to produce the PDMS templates. (b) Protocols used to produce the patterned: (*i*) agarose, (*ii*) PDMS, and (*iii*) gelatin.

An overview of the fabrication protocols to produce the patterned materials is provided in Fig 1, with accompanying procedures explained in the following sub-sections. In brief, a negative-tone photoresist mold master was fabricated using standard soft-lithography processes. The mold master comprised of an array of circular pillars, with a height of 5 μm and a diameter of 15 μm. From this mold master the PDMS templates were produced. In turn, the PDMS templates were used to fabricate patterned agarose, PDMS, and gelatin substrates. The patterned materials were then used in our investigation towards finding a suitable replica leaf surface material. In this process the mold master and PDMS template were representative of a living leaf surface and leaf imprint, respectively, and the patterned materials were representative of a replica leaf [28–31]. We selected PDMS as our template material as PDMS is well-established in microfabrication and is routinely used in double-casting. In addition, PDMS has been shown to exhibit nanoscale pattern resolution [41, 42].

### 2.1 Mold master fabrication

For the mold master substrate a 4” prime grade silicon wafer was used. The wafer was dehydrated at 185°C overnight in an oven to improve photoresist adhesion. The wafer was then removed from the oven and left to cool to room temperature. Subsequently, the wafer was cleaned in oxygen plasma for 10 minutes. ADEX05^TM^ (DJ MicroLaminates), an epoxy dry film photoresist, was then processed to produce the mold master [39]. Prior to casting the PDMS template, the mold master was treated with Trichloro(1H,1H,2H,2H-perfluorooctyl)silane (448931, Sigma-Aldrich) for two hours. This treatment was undertaken to facilitate the removal of the PDMS template.

### 2.2 PDMS template fabrication

The PDMS template was produced using standard replica molding techniques (Fig 1A) [45]. The two-part elastomer PDMS (Sylgard 184, Dow Corning) was prepared at a ratio of 10:1 w/w (base to curing agent) following the manufacturer guidelines. In summary, the base and curing agent were thoroughly mixed together in a plastic cup, and then placed into a vacuum desiccator (Z119016, Sigma-Aldrich) to remove the air bubbles. Once all the air bubbles disappeared, the PDMS was poured onto the mold master and degassed again to remove any introduced air bubbles. Once all the air bubbles disappeared, the PDMS and mold master were placed on a hot plate for two hours at 80°C to allow the PDMS to set. Once set, the PDMS template was carefully removed from the mold master. The PDMS template was then placed on a hotplate for a further two hours at 80°C to improve the durability of the template.

Several templates were fabricated to produce enough patterned samples for all experiments. All patterned material substrates were fabricated to an overall height of 3 mm. This was achieved by using rings with an internal diameter of 66 mm and filling the ring to the 3 mm line. From the patterned material substrates smaller samples were taken using a cork borer (Usbeck, Germany). Samples with a diameter of 7.8 or 11.5 mm were used in the experiments.

### 2.3 Patterned agarose fabrication

In preparation of casting the patterned agarose, the PDMS templates were placed under vacuum for two hours. The agarose (Agarose Low EEO, A0576, Sigma-Aldrich) was added to phosphate buffered saline (PBS, P4417, Sigma-Aldrich) to produce concentrations of 2.5, 5, or 7.5% w/v. The agarose solution was then placed on a hot plate at 200°C until the agarose was fully dissolved–approximately 15 minutes. Once the powdered agarose was fully dissolved, the solution was then poured onto the degassed PDMS template. This stack was then placed in a vacuum desiccator (Z119016, Sigma-Aldrich) for ten minutes with an open outlet valve (Fig 1B(*i*)). Following this, the stack was left at room temperature to allow for the agarose to set for an hour. Once set, the patterned agarose was carefully peeled off the PDMS template [35, 46].

### 2.4 Patterned PDMS fabrication

In preparation for casting the patterned PDMS, the PDMS template was treated with 0.1% w/v hydroxypropylmethylcellulose (HPMC, H8384, Sigma-Aldrich) in a phosphate buffer saline (PBS, P4417, Sigma-Aldrich) for 10 minutes. After 10 minutes the PDMS was removed from the HPMC solution and promptly rinsed with deionized water, and dried thoroughly with nitrogen gas [47, 48]. The patterned PDMS was prepared at ratios of 5:1, 10:1, or 20:1 w/w (base to curing agent). The base and curing agent were thoroughly mixed together and the mixture was then degassed until no bubbles remained (Fig 1B(*ii*)). Following this, the PDMS was poured onto the PDMS template and degassed again. Once no bubbles remained, this stack was placed on a hot plate for two hours at 80°C to allow the patterned PDMS to set. Once set, the patterned PDMS was carefully removed from the PDMS template.

### 2.5 Patterned gelatin fabrication

Gelatin from porcine skin (gel strength 300, G2500, Sigma-Aldrich) was added to PBS (P4417, Sigma-Aldrich) to produce concentrations of 10, 12.5, or 17.5% w/v. The gelatin solution was then placed on a hot plate at 50°C until the gelatin was fully dissolved–approximately 30 minutes (Fig 1B(*iii*)). The microbial transglutaminase (mTG, Ajinomoto Co., Inc., activity of approximately 1000 U g^-1^) solution was prepared in 1 mL of PBS. The mTG solution was prepared to a final concentration of 10 U of mTG per one gram of gelatin. Then the mTG solution was thoroughly mixed to ensure the mTG was fully dissolved. Once dissolved, the mTG solution was added to the gelatin solution and quickly mixed together. Once thoroughly mixed, the gelatin-mTG solution was immediately poured on the PDMS template and left at room temperature for five minutes to stabilise. This stack was then placed into an oven at 37°C for four hours to set the gelatin. To enable the gelatin to be readily peeled off the PDMS template, the stack was removed from the oven and placed in a fridge at 4°C for 30 minutes. The patterned gelatin was then carefully peeled off the PDMS template [44].

### 2.6 Optical profiles

All optical profiles of the patterned material samples were obtained within 30 minutes after peeling from the PDMS template. This was done to minimise potential effects from degradation due to the ambient conditions in the laboratory. All 3D optical profiles were obtained using a Profilm3D optical profilometer (Filmetrics Inc., USA), equipped with a 20 × objective (CF Plan 20×/0.40 DI, Nikon). For agarose and gelatin the high sidewalls of the pillars made imaging difficult due to light scattering. To correct for this, the data was processed using the inbuilt *remove outliers* function in the Profilm3D software 2018 (ver. 3.2.7.2, Filmetrics Inc., USA). For the *remove outliers* function, the *invalid pixels filled in* method was used and the *maximum slope* was set to 10. During post processing, all images were filtered using a 3-point level function in Profilm3D software. This was done to account for any non-level placement of the samples on the Profilm 3D stage

### 2.7 Atomic force microscopy images

Atomic force microscopy (AFM) images were obtained for the mold master, PDMS template, and patterned PDMS. AFM images were not possible for agarose and gelatin due to the patterned agarose and gelatin degrading during the required imagining period, and the AFM tip losing contact. All AFM images were taken using a Digital Instruments Dimension 3100 (Vecco, USA) equipped with TAP300-G tips (BudgetSensors, USA) operating in tapping mode. All AFM images were analysed using Gwyddion (Version 2.49).

### 2.8 Degradation measurement method

Two conditions were examined to test the degradation of the patterned materials: (*i*) 30°C at a relative humidity of 25%, and (*ii*) 30°C at a relative humidity of 75%. A temperature of 30°C was selected as it sustains bacterial life. The humidity levels were selected to: (*i*) mimic dry conditions similar to a climate-controlled laboratory; and (*ii*) a higher humidity that would slow the degradation of the patterned samples (prepared as detailed in the following sub-section). Five samples per the three different concentrations for each material were used. Each sample had an initial diameter of 11.8 mm. Weight measurements were taken at 0, 2, 4, 6, 12, 24, 48, and 72 hours. A weightless percentage measure was then determined for each of the time points. Results are presented as mean ± SEM (standard error of the mean).

### 2.9 Humidity

The degradation of agarose and gelatin is undesirable for investigating microorganisms in the phyllosphere. Consequently, an environment was sought to minimise the degradation of the materials. This environment must be favourable for microorganisms, and minimise any potential swelling of the materials. To achieve this, a saturated salt solution was prepared by dissolving 72 g of sodium chloride (71382, Sigma-Aldrich) in 200 mL of deionised water. The saturated salt solution was then placed next to the patterned material samples in a 7 L airtight container. Following this, the airtight container was placed in an oven at 30°C, which resulted in a constant relative humidity of 75% (see S1 Fig for humidity data) [49–51].

### 2.10 Contact angle measurement with water method

All contact angle measurements were undertaken with a CAM200 (KSV Instruments Ltd, Finland) integrated with KSV CAM Optical Contact Angle and Pendant Drop Surface Tension Software (ver. 4.01, KSV Instruments Ltd, Finland). Flat samples were compared against patterned samples for each material at the three selected concentrations. Five samples with a diameter of 11.8 mm each were measured for each concentration. This diameter was selected to minimise the potential occurrence of edge effects on the water droplets. For agarose, which is considerably hydrophilic, all results presented are for droplets with a volume of less than 40 μL. In contrast, for PDMS and gelatin, water droplets with a volume less than 60 μL were analysed. Prior to the contact angle measurements, all samples were dried with dry nitrogen gas to minimise any potential effects from surface moisture. In addition, experiments were conducted within an hour of peeling the material off the PDMS template. The surface energy of all the materials was determined using deionised water.

Results are presented as mean ± SEM. For statistical analysis, Student’s t-test or ANOVA was performed using GraphPad Prism 7 (GraphPad Software, USA). P values less than 0.05 were considered significant (*P<0.05, **P<0.01, ***P<0.001, and ****P<0.0001).

### 2.11 Bacteria culture protocol

*Pantoea agglomerans* 299R, a model leaf colonising bacterium that was previously isolated from a healthy leaf of a pear tree, was grown overnight on nutrient agar plates (13 gL^-1^ Lysogeny broth and 15 gL^-1^ bacteriological Agar, Oxoid) at 30°C [52]. The *P*. *agglomerans* was then harvested using a sterile inoculation loop and resuspended in 5 mL of sterile phosphate buffer (8 gL^-1^ NaCl (LabServ), 0.2 gL^-1^ KCl (LabServ), 1.44 gL^-1^ Na_2_HPO_4_-GPR (AnalaR), and 0.24 gL^-1^ KH_2_PO_4_ (AnalaR), pH 7.4). Following this, bacteria were washed by centrifugation at 1150 RCF for five minutes at 10°C. The supernatant was discarded and the bacteria was suspended in fresh phosphate buffer to an OD_600 nm_ of 0.2, corresponding to approximately 2 x 10^8^ bacteria per mL.

### 2.12 Bacteria viability protocol

Samples with a diameter of 7.8 mm were used for the bacteria viability experiments. Five samples were taken per time point and all samples were sterilised for 15 minutes with ultra-violet sterilisation. Then 100 μL of the bacterial solution (~2 x 10^7^ bacteria) was inoculated to the patterned samples, using an air brush (KKmoon T-180 Airbrush, China) at 1×10^5^ Pa [53]. Patterned samples coated with bacteria were then incubated at 30°C at a relative humidity of 75%.

Five samples were randomly selected per measurement time points corresponding to 0, 2, 4, 6, 12, 24, 48, and 72 hours. Each individual sample was suspended in 1 mL of fresh phosphate buffer in a 1.5 mL Eppendorf tube. The individual samples were then placed in a Bead Ruptor 24 (Omni 15 International Inc., USA) bead mill homogenizer and vortexed at 2.6 ms^-1^ for five minutes. The samples were left to cool for five minutes to minimise any potential thermal stress and then vortexed for another five minutes. Following this, the samples were placed in an ultrasonic bath (E Easy Elmasonic, Elma Schmidbauer GmbH, Germany) for five minutes. For each sample, 100 μL of supernatant was recovered [54, 55]. The recovered supernatant was then plated onto nutrient agar by undertaking serial dilutions using PBS. At each dilution step 10 **μL** aliquots were taken, with a final dilution of 10^−6^ being plated. Colony-forming units (CFU) were expressed as bacterial cell density per unit area for each sample (7.8 mm diameter). The cell density was normalised to the initial number of cells after inoculation. This was undertaken to enable a comparison between the survival curves of the bacterial colonies on the different materials. Results are presented as mean ± SEM

## 3 Results and discussions

### 3.1 Pattern resolution

The microstructures found on leaves are dependent on the plant species and can range in complexity. Microstructures found on leaves include groves and stomata (pores) to the more complex structure of trichomes (leaf hairs). The ability to sufficiently replicate the surface topography of a living leaf is important for producing replica leaf surfaces that can be used as a control surface in phyllosphere microbiology. This is of significance, as the topography of the leaf surface has the ability to influence colonisation and attachment behaviours of microorganisms [4, 20].

**Fig 2:**
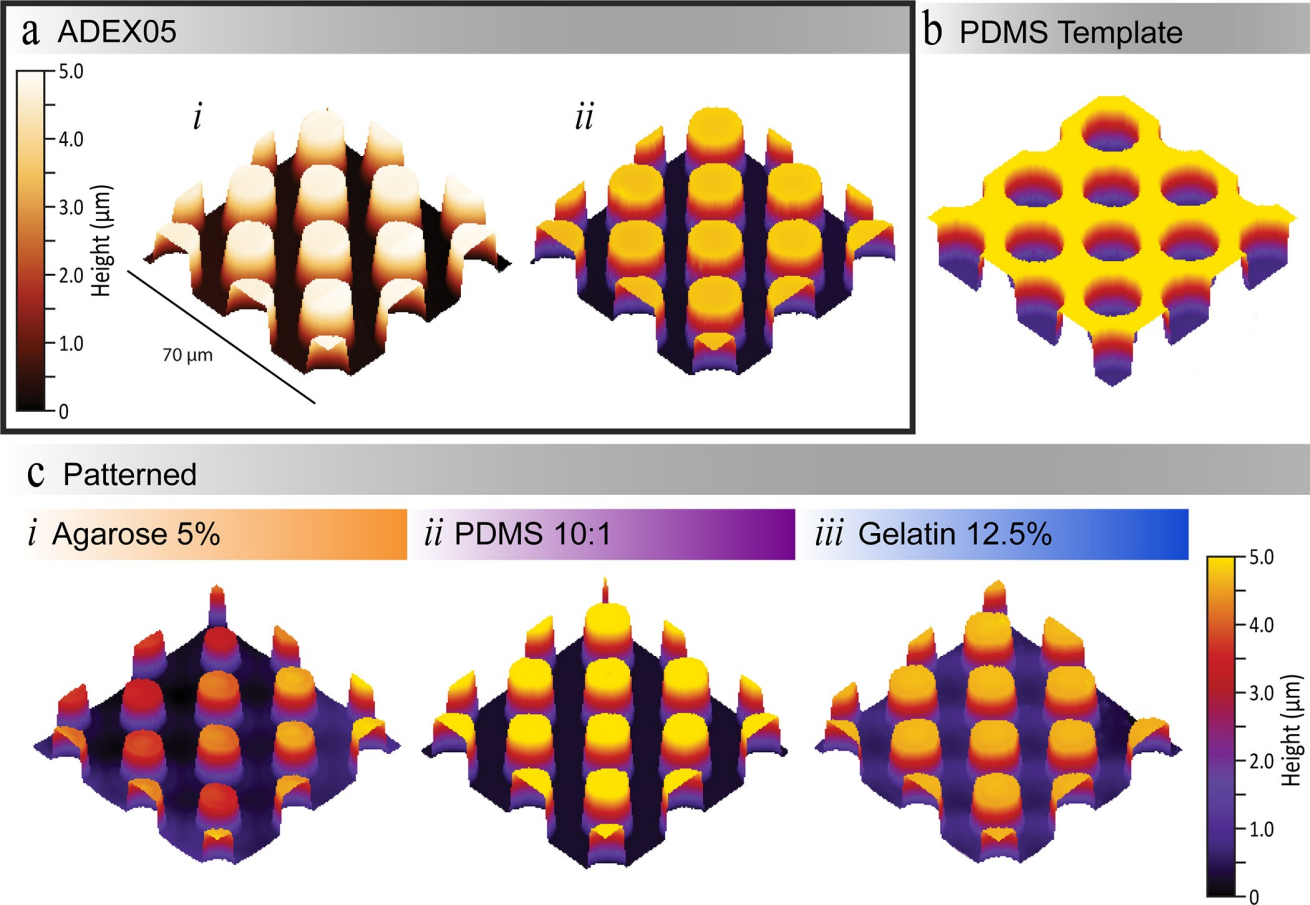
Resolution images. (a) Comparison of the photoresist mold master (ADEX05) undertaken by (*i*) AFM and an (*ii*) optical profilometer. (b) PDMS 10:1 template 3D optical profile. (c) Optical profiles for (*i*) agarose 5% w/v, (*ii*) PDMS 10:1 w/w, and (*iii*) gelatin 12.5% w/v. All optical profilometer images were filtered using a 3-point level function in Profilm3D Software (Filmetrics). See S2 Fig. for AFM and optical profilometer image comparison for the patterned PDMS replication process.

To investigate the capability of our selected materials to replicate the complex topography of leaves, we used a test pattern. The test pattern consisted of circular pillars with a height of 5 μm and a diameter of 15 μm. Such dimensions are comparable to the features found on leaf surfaces–trichome, stomata, and grooves. For example, *Pelargonium zontal* stomata have a length and width of 16 μm by 9 μm [56]. In contrast, trichomes on a tomato leaf have a width of approximately 17 μm at their base [20]. Resolution comparisons of the patterned materials were undertaken using a 3D optical profilometer (Fig 2). Optical profiles were selected over more commonly used techniques to compare microstructure resolution, such as AFM and scanning electron microscopy (SEM). The use of these techniques was hindered by the softness and quick degradation of agarose and gelatin. When investigating the suitability of AFM imaging, we regularly observed the AFM tip losing contact with the sample during imaging. The use of SEM imaging was limited by the requirement of a conductive coating, as both agarose and gelatin are non-conductive. To produce a conductive coating, the samples would have to be placed under vacuum. However, tests with a short three-minute vacuum resulted in a noticeable structural degradation in both materials. Conversely, obtaining a 3D optical profile requires no modifications to be made to the materials, and images can be acquired in approximately three minutes; thus, minimising any degradation affects (see section 2.6 Optical profiles for more information).

To prove the validity of optical profilometry for our materials we compared our results to AFM scans undertaken on the mold master, PDMS template and the patterned PDMS (see S2 Fig and S1 Table). The measurements obtained using the AFM and optical profilometer were in agreeance (Table 1 and S1 Table). For example, the height of the mold master was measured to be 4.50 ± 0.03 μm using the optical profilometer, which lies in the range measured by the AFM of 4.44 ± 0.16 μm. Furthermore, the measured widths were 14.64 ± 0.47 μm and 14.94 ± 0.21 μm for images taken by the AFM and optical profilometer, respectively. In consequence, this indicates that no significant difference existed between 3D profiles taken by either AFM or an optical profilometer.

**Table 1:** Pattern resolution measurements. Height and width measurements were taken from 20 pillars (or wells, as was the case from the PDMS template). All data is represented as mean ± standard deviation. Standard deviation has been used to show the scattering in measured values. See S2 Fig and S1 Table for AFM and optical profilometer image comparison for the PDMS patterned replication process.

	AFM	Optical Profilometer				
	ADEX05 Mold Master	ADEX05 Mold Master	PDMS Template	Patterned Agarose	Patterned PDMS	Patterned Gelatin
**Height (μm)**	4.44 ± 0.16	4.50 ± 0.03	4.18 ± 0.01	2.96 ± 0.60	4.71± 0.01	3.89 ± 0.06
**Width (μm)**	14.64 ± 0.47	14.94 ± 0.21	15.11 ± 0.52	10.01 ± 0.46	14.00 ± 0.38	13.50 ± 0.48

A summary of the optical profilometry measurements of the height and width of the pillars is presented in Table 1, or in the case of the PDMS template, the depth and width of the well. Twenty pillars were measured for each dimension measurement.

In the case of agarose the pillars were smaller by 1.54 ± 0.57 μm (in height) and 4.93 ± 0.25 μm (in width) in comparison to the mold master (Table 1). In addition, a larger variation in measured heights for agarose was observed. We conjectured this variation was most likely a result of non-uniform shrinkage occurring while the agarose sets during fabrication [57].

In addition, pillars produced in gelatin were smaller. The height and width of the pillars in gelatin were 0.61 ± 0.03 μm and 1.14 ± 0.27 μm smaller than the mold master, respectively (Table 1). We conjectured that this was due to the gelatin degrading while setting. In addition, we observed that pillars regularly broke when peeling the gelatin off the PDMS template. We minimised the occurrence of the pillars breaking by following the recommendations made by Paguirigan and Beebe, and placed the gelatin and PDMS template in a fridge prior to peeling the gelatin off [44]. Furthermore, from our experience the trichomes (leaf hairs) would not withstand being peeled off the PDMS template even following this protocol, as they are inherently fragile [20].

Conversely, the pillars replicated into PDMs were more representative of the mold master. The dimensions of the pillars produced in PDMS were measured to be within 10% of the pillars of the mold master, whereas, pillars produced in agarose and gelatin differed by more than 10%. This indicates, that from a pure pattern replication perspective, a replica leaf made from PDMS would be more representative of the living leaf topography, than a replica made from gelatin or agarose.

### 3.2 Degradation measurements

Microbiology experiments can take anywhere from a few minutes or hours to days and even weeks. Consequently, a replica material should not degrade during the duration of the experiment as this will result in moisture and/or topography changes, which influence microorganism behaviour [6, 13]. Topography changes, in particular, may prevent certain experiments from being undertaken, such as studies investigating the influence of chemicals or air quality [4].

**Fig 3:**
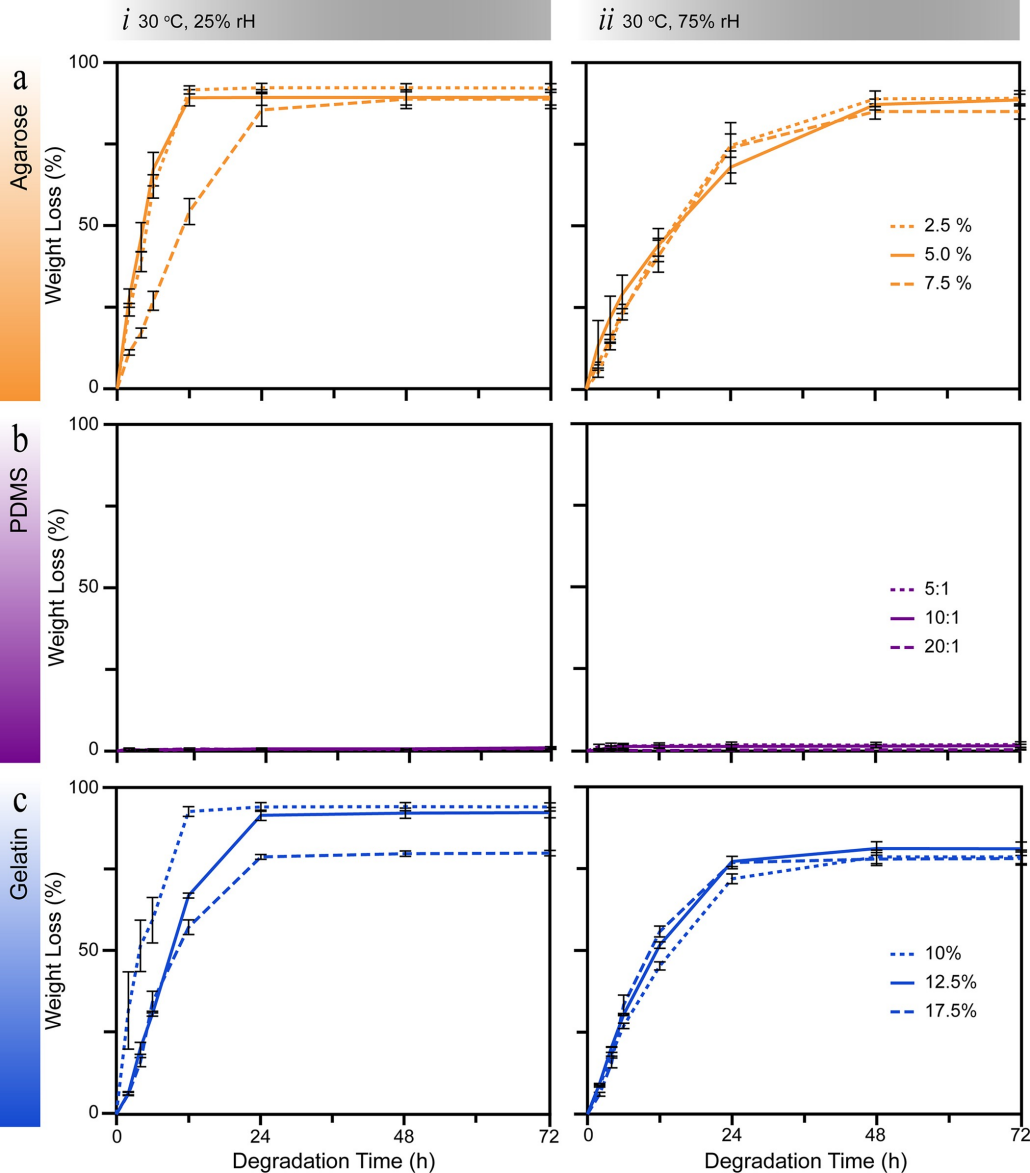
Degradation measurements for the three selected materials. The weight loss over 72 hours for (a) agarose, (b) PDMS, and (c) gelatin was investigated at relative humidities (rH) of (*i*) 25% and (*ii*) 7 5% at 30°C. Five samples were measured for each material at each concentration.

To study material stability, we examined the degradation of the patterned materials over three days under two humidity conditions at 30°C (Fig 3). A temperature of 30°C was selected as it is a common temperature to cultivate environmental bacteria. A relative humidity of 25% was selected to mimic the humidity often found in dry conditions. Conversely, a relative humidity of 75% was selected to slow the degradation of the patterned samples. Furthermore, these two relative humidities can be found in cities around the world [58].

For patterned agarose a relative humidity of 75% effectively slowed the degradation to the equilibrium point by 36 hours (Fig 3A). We defined the equilibrium point as the time in which the material reaches an equilibrium moisture exchange with the surrounding environment. Regardless of the concentration of agarose, the equilibrium point (88 ± 1% weight loss) was reached within 24 and 48 hours for 25% and 75% relative humidity, respectively. No significant difference in the degradation characteristics was observed in a relative humidity of 75%. This indicated that there is no advantage in changing the concentration of agarose–between 2.5 and 7.5% [31, 35].

In contrast, for gelatin a relative humidity of 75% lowered the equilibrium point (Fig 3C). Furthermore, regardless of the concentration of gelatin the equilibrium point (80 ± 1% weight loss) was reached within 24 hours in both humidity conditions. Similar to agarose, changing the concentration of gelatin in PBS did not improve the degradation characteristics. For patterned PDMS no degradation was observed over the 72 hours experimental duration in either humidity conditions and no concentration dependence was observed (Fig 3B).

In summary, the degradation results indicate that a replica leaf produced from PDMS would be stable in environmental humidities that plants occupy around the world. Whereas, agarose and gelatin degrade within 48 hours of fabrication in environmental humidity ranges. In literature, agarose and gelatin have been stored in water or in a fridge to minimise degradation, respectively [44, 46]. However, these conditions are not compatible with phyllosphere microbiology experiments and also affect the resolution of the replicated microstructures. For instance, storing agarose in water results in swelling and hence changes the microstructure topography. Furthermore, the majority of microorganisms that reside on plants, generally do not grow in low temperatures such as those experienced in a fridge– 0 to 4°C. On the other hand, replica leaves produced in PDMS would allow samples to be sent to laboratories on the other side of the world. As PDMS replica leaves would not degrade in the typical environmental conditions experienced during transportation. Unless stored in the aforementioned unfavourable conditions, transportation would quickly liquefy agarose and gelatin replica leaves.

### 3.3 Contact angle measurements

**Fig 4:**
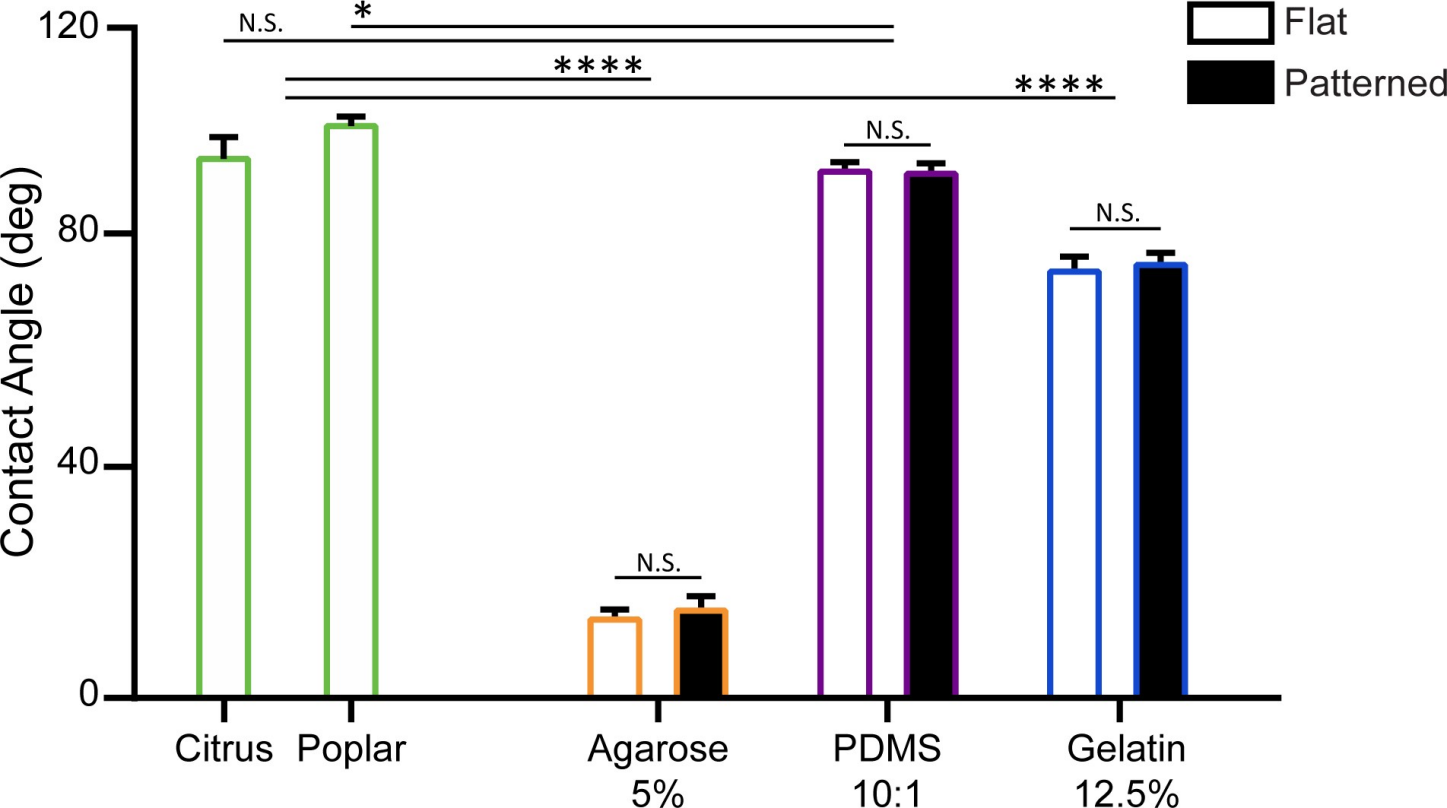
Contact angle comparison of leaf cuticles with the three selected replica materials. Contact angles of bitter orange and poplar leaf cuticles, are compared with contact angles of agarose 5% w/v, PDMS 10:1 w/w and gelatin 12.5% w/v. See S3 Fig for contact angle measurements for the three concentrations of each material. Data is presented as the mean ± SEM, *P<0.05, **P<0.01, ***P<0.001, and ****P<0.0001. N.S. indicates no significant difference between measurements.

The hydrophobicity of a leaf surface is another important property that needs to be considered when selecting a suitable replica material. As the presence of water on a leaf surface impacts resource availability and colonisation patterns of microorganisms in the phyllosphere. Furthermore, the hydrophobicity of the leaf surface influences the microorganism attachment processes. Microorganisms can achieve surface attachment by adapting to enable attachment or by forming biofilms [13, 59]. In the context of surface energy, a surface is classified as either hydrophilic, hydrophobic or superhydrophobic when the contact angle of water is < 90°, > 90°, and >150°, respectively. For comparison, we selected enzymatically isolated leaf cuticles from *Citrus × aurantium* (bitter orange) and *Populus × canescens* (poplar) plant species (Fig 4)[23]. In addition, we compared both flat and patterned surfaces for each of the three replica materials (see S3 Fig, for contact angles at different material concentrations).

Our results indicated that the patterned PDMS (95.5 ± 0.6°, N = 5) was hydrophobic, and the patterned agarose (15.9 ± 0.9°, N = 5) and gelatin (78.3 ± 1.0°, N = 5) were hydrophilic. These results are in agreeance with literature [41, 60]. Furthermore patterning the different materials with our test pattern did not influence the hydrophobic properties of the materials (Fig 4). This was attributed to the size of the pillars, as there is no change in the pinning of the water droplets on this scale [61]. In addition, changing the concentration of the materials did not change the hydrophobicity of the materials (S3 Fig).

For comparison, isolated bitter orange and poplar leaf cuticles used, and they were determined to be hydrophobic, with contact angles measured to be 97.9 ± 2.7° (N = 5) and 103.8 ± 0.7° (N = 5), respectively. In general, contact angles of plant leaves can vary considerably from hydrophilic to superhydrophobic [32, 62, 63]. In our case, no significant difference in contact angle was measured between bitter orange and PDMS. However, a slight difference was measured between Poplar and PDMS (P < 0.05, N = 5). Nonetheless, these results provide a significant contrast to the difference observed between the leaf cuticles and both agarose and gelatin (Fig 4).

In summary, the hydrophobicity of PDMS was comparable to bitter orange and poplar. Whereas, agarose and gelatin were considerably more hydrophilic. Thus indicating PDMS is a suitable replica surface material for conducting attachment studies of hydrophobic leaves. In addition, the degree of hydrophobicity of PDMS can be modified temporarily through oxygen plasma or extended in duration with polyvinylpyrrolidone (PVP) treatment. Both modifications are not harmful to microorganisms [64]. The use of PVP treatment for example, would enable more extensive attachment studies to be undertaken using a PDMS replica surface. In literature this has been highlight as an area that requires more extensive studies to be undertaken [13].

### 3.4 Bacterial survival

**Fig 5:**
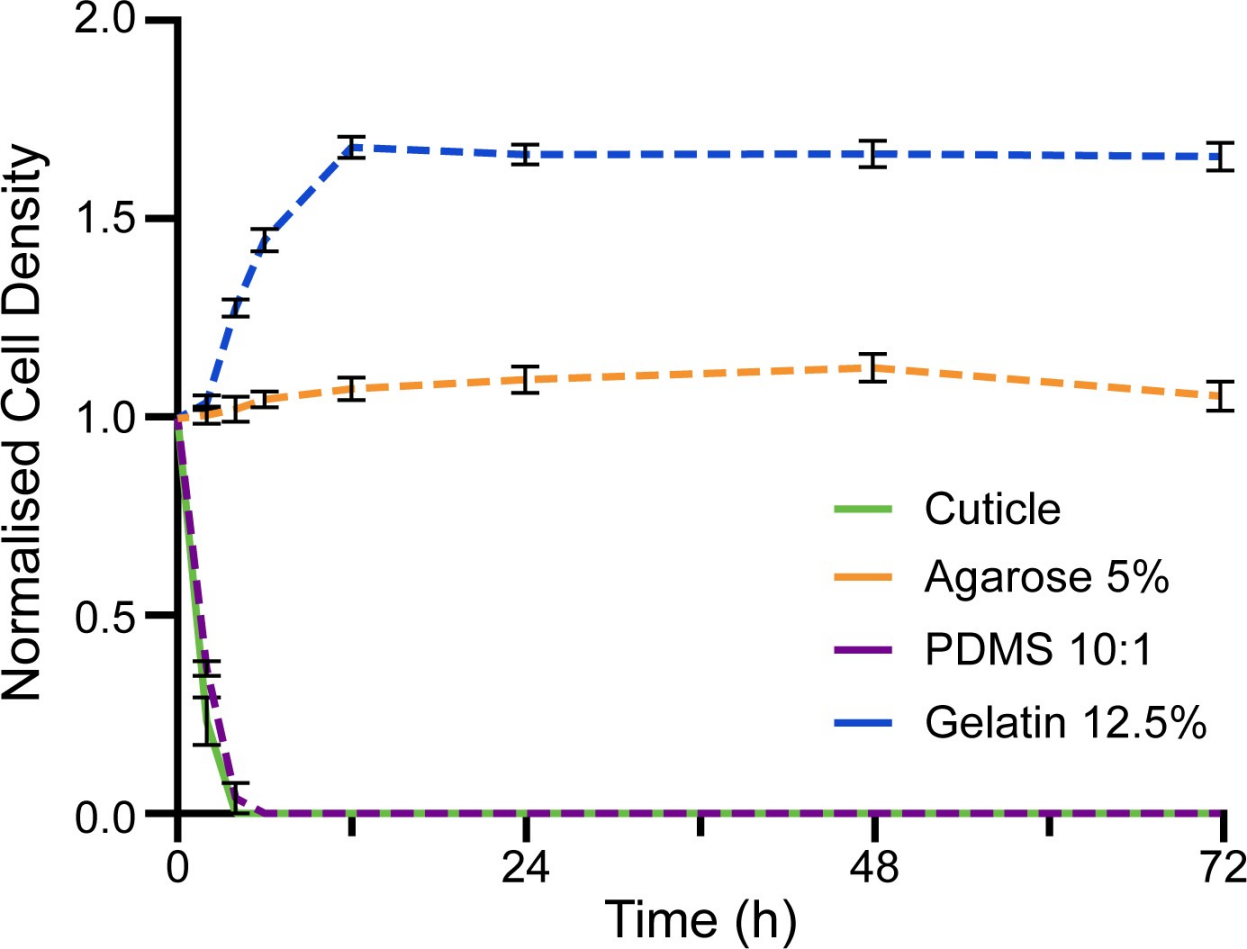
Normalised cell density of *Pantoea agglomerans* 299R. The normalised cell density of *P*. *agglomerans* 299R was analysed over 72 hours on: *Citrus* × *aurantium* (bitter orange) cuticles, agarose 5%, PDMS 10:1 w/w, and gelatin 12.5%. See S4 Fig for contact normalised cell density measurements for the three concentrations of each material. Data here is presented as the mean ± SEM.

To assess the suitability of the materials to support microorganism life we conducted bacterial survival experiments, with *Pantoea agglomerans* 299R as our model microorganism (Fig 5). The bacterium *P*. *agglomerans* 299 was isolated from a Bartlett pear tree leaf. Strain *P*. *agglomerans* 299R is a spontaneous rifampicin resistant mutant of *P*. *agglomerans* 299 [52]. We selected *P*. *agglomerans* 299R as our model microorganism as it is: (*i*) a model microorganism for leaf colonisation, (*ii*) well characterised and fully sequenced, and (*iii*) it is genetically amendable (able to produce mutants and bioreporters)[27, 52]. We compared the bacterial survival characteristics from the replica materials against isolated *Citrus × aurantium* (bitter orange) leaf cuticles. We used isolated leaf cuticles, as we wanted to determine which material closely resembled the bacterial survival characteristics observed on the surface of leaves, in the absence of the nutrients supplied from the leaf. This was important, as we were looking for a suitable material to form a replica leaf platform for phyllosphere microbiology studies.

In the case of agarose, the population of *P*. *agglomerans* 299R stabilised at a normalised cell density of 1.06 ± 0.03 (N = 5) after 24 hours (Fig 5), regardless of whether the material was patterned or flat (S4 Fig). We conjectured that the population stabilised due to the presence of moisture on the surface of the agarose, as bacterial life can be sustained when moisture is present in the environment. In this case the moisture on the surface of the agarose was a result of the agarose degrading. Furthermore, agarose is a potential source of nutrients for bacteria, due to agarose being derived from red seaweed which is comprised of polysaccharides. However, *P*. *agglomerans* 299R do not have the necessary enzymes to hydrolyse the α-(1 → 3) and β-(1 → 4) glyosidic bonds between the material monomers [65]. Thus, we conjectured that the population of *P*. *agglomerans* 299R did not increase on agarose due to the lack of nutrient supply. Conversely, on gelatin an increase in the population of *P*. *agglomerans* 299R was observed in the first 12 hours after inoculation (Fig 5). This growth was attributed to gelatin providing a nutrient source in the form of peptides and proteins. As the bacterial enzymes were able to hydrolyse the peptide bonds, gelatin provided a suitable nutrient source of carbon and nitrogen. After 12 hours the population stabilised at a normalised cell density of 1.69 ± 0.03 (N = 5). Thus, indicating that this is the largest population density gelatin can sustain. It is important to note that the enzymatic breakdown of gelatin as a nutrient source by the bacteria would influence the degradation characteristics of the material [66]. After all, this breakdown often results in gelatin becoming liquefied [43].

In contrast, *P*. *agglomerans* 299R populations were not sustained on either isolated bitter orange cuticles or PDMS. Once a cuticle is isolated from a leaf, there is no nutrient support from the leaf and the cuticle itself does not provide any nutrients for the *P*. *agglomerans* 299R. Hence, no increase in the population of *P*. *agglomerans* 299R was observed. Furthermore, with the absence of moisture on the surface of the bitter orange cuticles, the *P*. *agglomerans* 299R died within six hours (Fig 5). In the case of PDMS, bacteria do not have the enzymes necessary to degrade the PDMS to form a sustainable nutrient source. PDMS also did not degrade in the experimental conditions (Fig 3), indicating that an exchange of moisture with the environment did not occur. As a result, PDMS did not provide the necessary moisture to sustain bacterial life. The lack of nutrients and moisture resulted in the population of *P*. *agglomerans* 299R dying within six hours of inoculation to the patterned PDMS surface. Which is comparable to the behaviour observed on the isolated leaf cuticles.

In summary, agarose and gelatin will provide a moisture source to sustain bacterial life. In addition, gelatin can be degraded by *P*. *agglomerans* 299R to provide a nutrient source that promotes an increase in population—until the maximum population sustainable by gelatin is reached. Thus, indicating that the inherent properties of agarose and gelatin influences bacterial viability. Which also influences the choice of replica leaf material that can be used for phyllosphere microbiology studies. For instance, any inherent nutrient source from the replica material would influence nutrient supply studies. Hence any results obtained from such a system would be deemed unreliable. Conversely, bacterial life was not sustained on either PDMS or bitter orange cuticles. This indicated that PDMS is more representative of a living leaf surface. In addition, through suitable modification PDMS could enable a controlled nutrient or moisture supply to be introduced. For example, his could be achieved with the use of fillers such as carbon nanotubes. This would enable a nutrient supply more representative of the living leaf, while retaining the physical advantages of PDMS [67].

## 4 Conclusions

Our work has demonstrated the potential of PDMS as a replica leaf material for phyllosphere microbiology. At the same time, our results highlighted the drawbacks of agarose and gelatin through comparing optical resolution, degradation characteristics, hydrophobic properties and bacterial survival to PDMS.

Using optical profilometry, we demonstrated that agarose and gelatin replicas would not provide topography comparable to that of a living leaf. Dimensions of the test pillars reproduced in agarose and gelatin were found to differ from the mold master by more than 10%. Furthermore, agarose and gelatin degraded considerably within 72 hours in both high and low humidity conditions at 30°C. On the other hand, the height and width of pillars reproduced in PDMS were within 10% of the dimensions measured for the mold master. PDMS also displayed no structural degradation within 72 hours. These results indicate that in terms of topography, a replica leaf made from PDMS would be more representative of a leaf surface. The suitability of PDMS was further supported by measured contact angles, which were comparable to those of isolated *Citrus × aurantium* and *Populus × canescens* leaf cuticles.

In addition, we examined the influence of the replica materials on bacterial survival in comparison with isolated *Citrus × aurantium* leaf cuticles. Both PDMS and the isolated leaf cuticles were unable to sustain bacterial life, thus indicating that PDMS is representative of a leaf cuticle. On the other hand agarose and gelatin were both able to sustain bacterial life, thus indicating that these materials would influence microorganism behaviour. As a result, both of these materials must be considered unsuitable for nutrient supply studies.

In summary, the results presented here indicate that in contrast to agarose and gelatin, the combined properties of PDMS make for a suitable replica material for phyllosphere microbiology. Our experiments highlight the importance of considering the inherent material properties when selecting a material as a replica surface. In our current work, we are investigating the preparation of biomimetic leaf replicas made from PDMS with tailored nutrient permeability for plant-microbe interactions at a single-cell resolution.

## Supporting information

S1 Fig Humidity experimental control experimental results. Experimental parameters detailed in section 2.8 Humidity.
(TIF)

S2 Fig AFM and optical image comparison. Comparison of the (a) photoresist mold master resolution; (b) PDMS 10:1 w/w template; and (c) patterned PDMS 10:1 w/w, undertaken by (*i*) AFM, and, an (*ii*) optical profilometer.
(TIF)

S3 Fig Contact angle comparisons on flat and patterned replica materials at three different concentrations. Contact angles for (a) agarose, (b) PDMS, and (c) gelatin.
(TIF)

S4 Fig Bacteria viability of *Pantoea agglomerans* 299R. Bacteria viability for bacteria at (*i*) different concentrations, for (a) agarose, (b) PDMS, and (c) gelatin. Comparison of (*ii*) bacteria viability on flat and patterned replica materials for (a) agarose 5% w/v (b) PDMS 10:1 w/w, and (c) gelatin 12.5% w/v.
(TIF)

S1 Table AFM and optical image comparison measurements. Height and Width Measurements were taken from 20 pillars (or wells, as was the case for the PDMS template). All data is represented as mean ± standard deviation. Standard deviation has been used to show the scattering in measured values.
(PDF)
