# Supplementary figures and images for: Comparison of replica leaf surface materials for phyllosphere microbiology
Source: PLoS One. 2019 Jun 6;14(6):e0218102. doi: 10.1371/journal.pone.0218102 (PMC6553772; doi:10.1371/journal.pone.0218102)

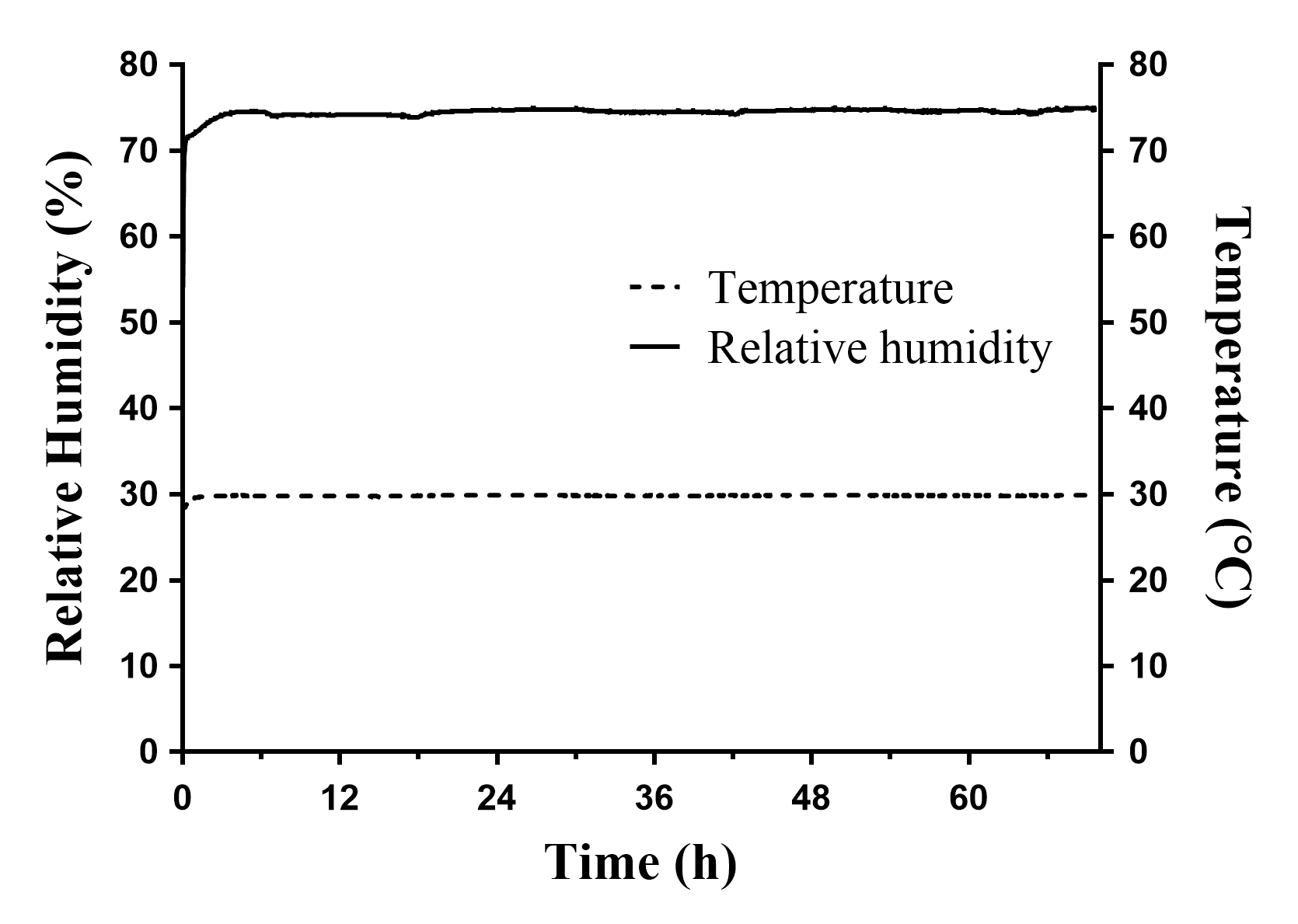

Supplement: S1 Fig — Experimental parameters detailed in section 2.8 Humidity. (TIF) [file pone.0218102.s001.tif]

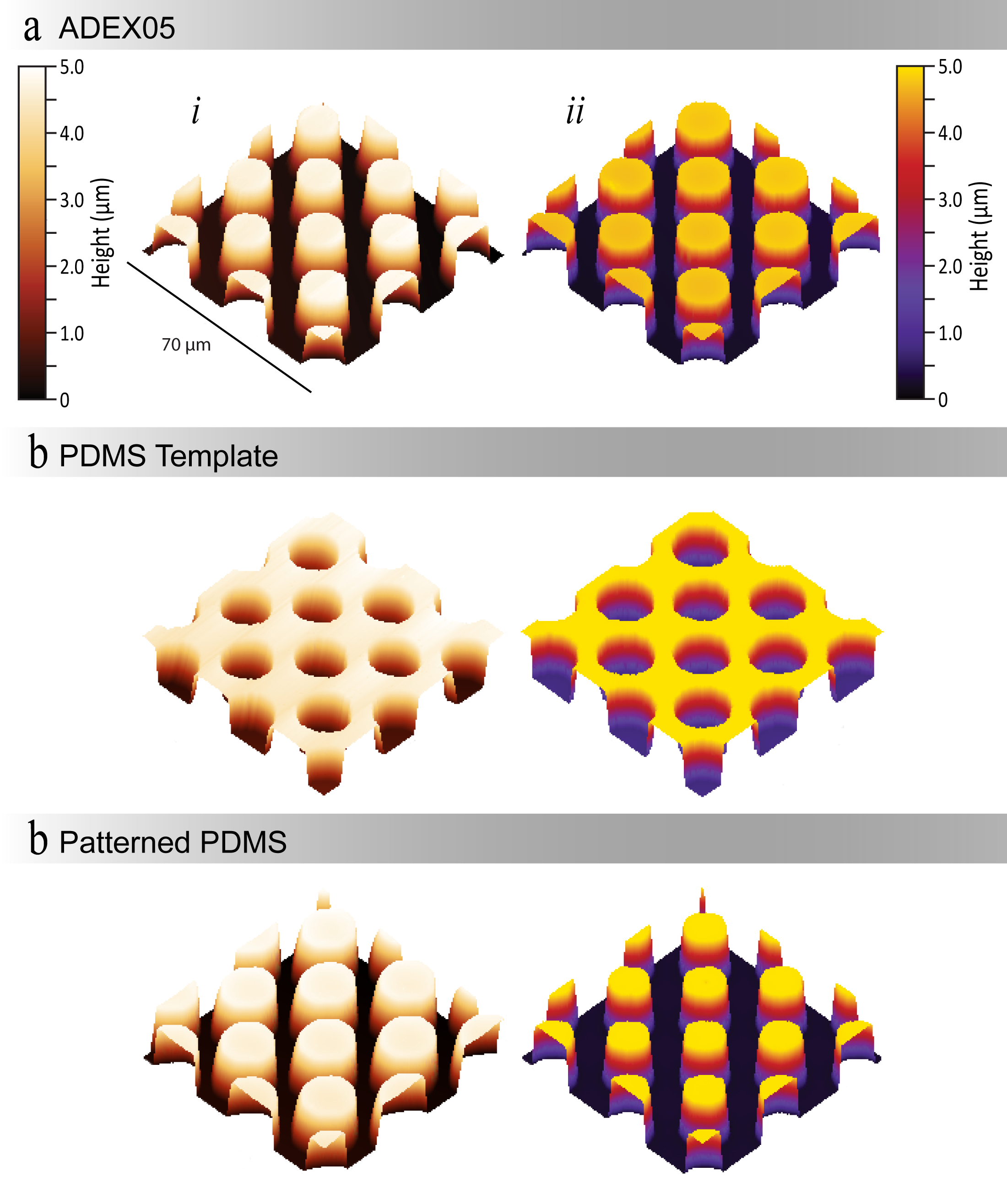

Supplement: S2 Fig — Comparison of the (a) photoresist mold master resolution; (b) PDMS 10:1 w/w template; and (c) patterned PDMS 10:1 w/w, undertaken by (i) AFM, and, an (ii) optical profilometer. (TIF) [file pone.0218102.s002.tif]

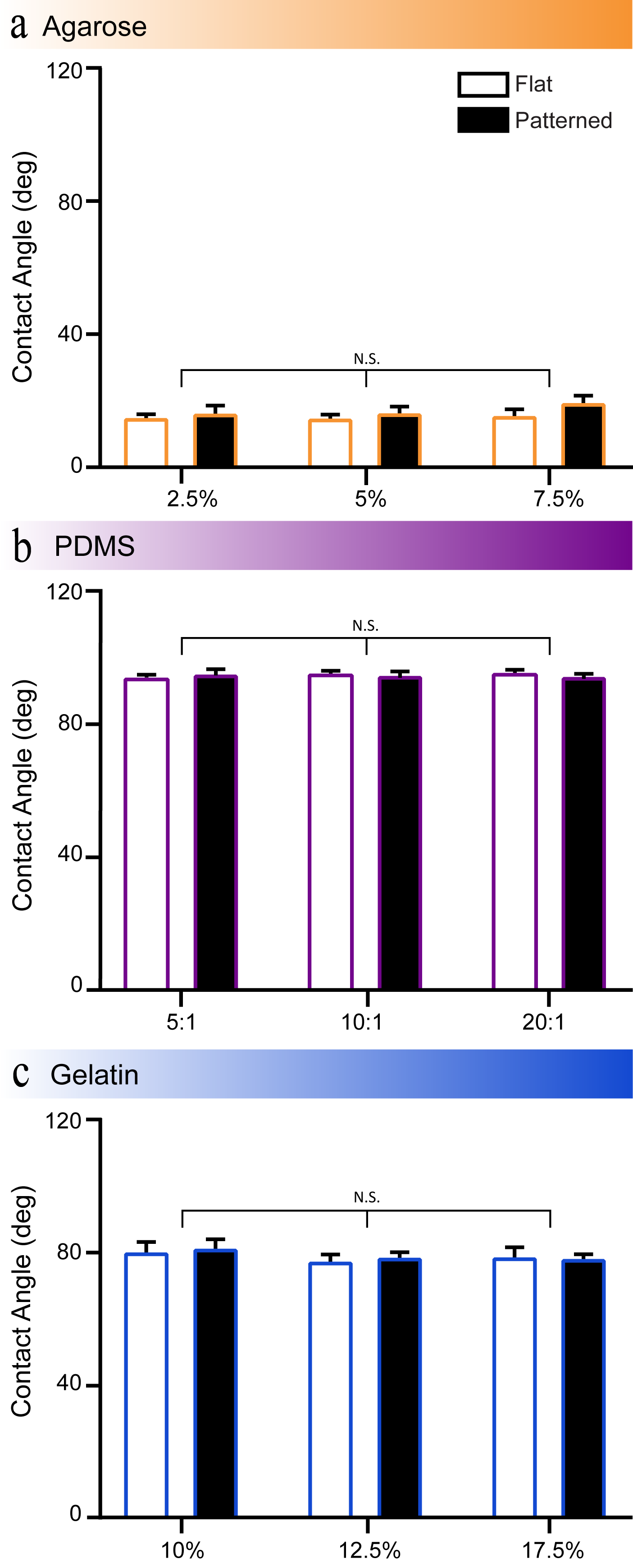

Supplement: S3 Fig — Contact angles for (a) agarose, (b) PDMS, and (c) gelatin. (TIF) [file pone.0218102.s003.tif]

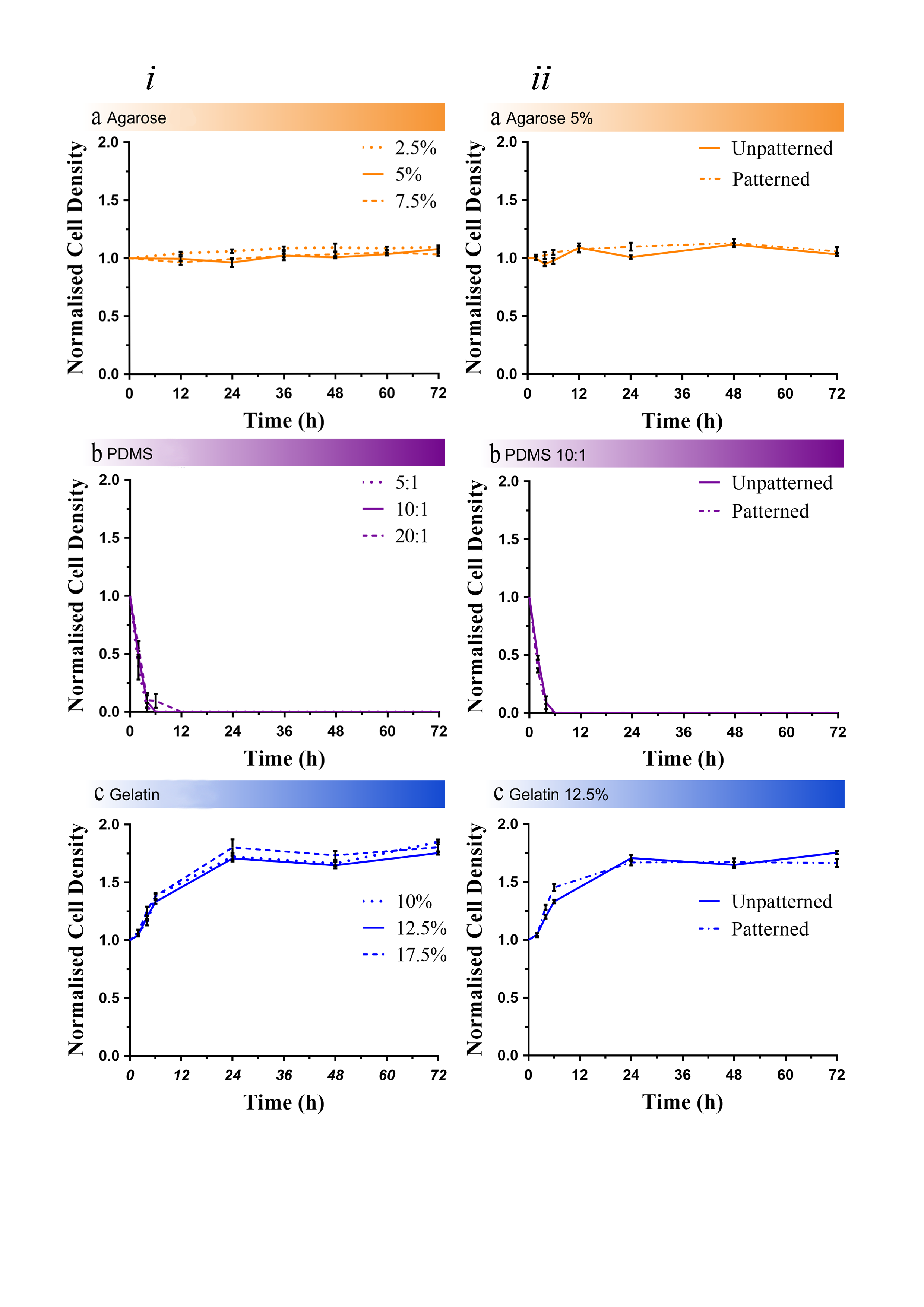

Supplement: S4 Fig — Bacteria viability for bacteria at (i) different concentrations, for (a) agarose, (b) PDMS, and (c) gelatin. Comparison of (ii) bacteria viability on flat and patterned replica materials for (a) agarose 5% w/v (b) PDMS 10:1 w/w, and (c) gelatin 12.5% w/v. (TIF) [file pone.0218102.s004.tif]
