# Supplementary material for: Comparison of replica leaf surface materials for phyllosphere microbiology
Source: PLoS One. 2019 Jun 6;14(6):e0218102. doi: 10.1371/journal.pone.0218102 (PMC6553772; doi:10.1371/journal.pone.0218102)
Supplement: S1 Table — Height and Width Measurements were taken from 20 pillars (or wells, as was the case for the PDMS template). All data is represented as mean ± standard deviation. Standard deviation has been used to show the scattering in measured values. (PDF) [file pone.0218102.s005.pdf]

S1 Table: AFM and Optical Image Comparison Measurements. Height and Width Measurements were taken from 20 pillars (or wells, as was the case for the PDMS template). All data is represented as mean ± standard deviation. Standard deviation has been used to show the scattering in measured values.

	**AFM**			**Optical Profilometer**		
	**Photoresist Mold Master**	**PDMS Template**	**Patterned PDMS**	**Photoresist Mold Master**	**PDMS Template**	**Patterned PDMS**
**Height (μm)**	4.44 ± 0.16	4.20 ± 0.06	4.58 ± 0.11	4.50 ± 0.03	4.18 ± 0.01	4.71± 0.01
**Width (μm)**	14.64 ± 0.47	15.23± 0.57	14.93 ± 0.32	14.94 ± 0.21	15.11 ± 0.52	14.00 ± 0.38
